# Editorial: Isolation, Modification, and Characterization of the Constituents (Cellulose, Hemicellulose, Lignin, et al.) in Biomass and Their Bio-Based Applications

**DOI:** 10.3389/fbioe.2022.866531

**Published:** 2022-05-13

**Authors:** Caoxing Huang, Chunlin Xu, Xianzhi Meng, Lei Wang, Xin Zhou

**Affiliations:** ^1^ Co-Innovation Center for Efficient Processing and Utilization of Forest Resources, College of Chemical Engineering, Nanjing Forestry University, Nanjing, China; ^2^ Laboratory of Natural Materials Technology, Åbo Akademi University, Turku, Finland; ^3^ Department of Chemical and Biomolecular Engineering, University of Tennessee, Knoxville, TN, United States; ^4^ College of Food Science and Engineering, Ocean University of China, Qingdao, China

**Keywords:** cellulose, hemicellulose, lignin, biotechnology, bio-applications

Lignocellulosic biomass is an abundant renewable biomaterial that is mainly composed of cellulose, hemicellulose, lignin, and extractives in different proportions (Figure 1). The constituents of cellulose and hemicellulose have been widely used as the precursors to prepare bio-materials, dietary fibers, and intermediates, which can be applied in the areas of biomedical therapy, food preservation, food additives, and cosmetics. Lignin is the most naturally abundant aromatic polymer that can be used to prepare bioactive components. Due to the natural recalcitrance of lignocellulosic biomass, an isolation approach should be carried out to obtain these constituents with high purity. In addition, various modification technologies are often applied to these constituents to improve their reactive activation or tailor the polymers with specific functions. Hence, seeking the appropriate approaches to isolation and modification is essential for the effective utilization of these biomass components.

**FIGURE 1:**
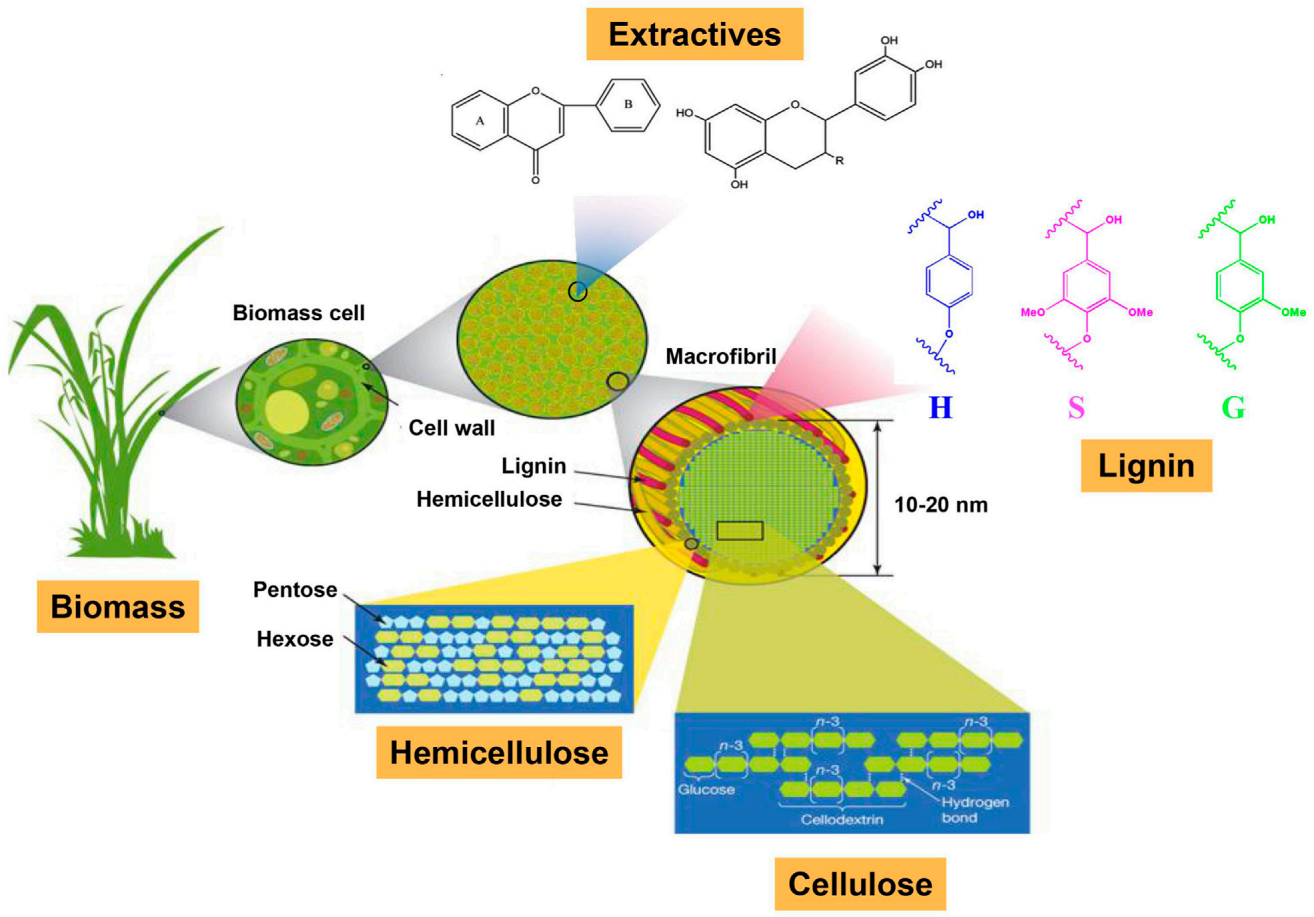
The major components in biomass.

To obtain the different constituents in biomass, physical, chemical, and combined methods have been widely investigated. Despite the tremendous progress that has been made in the development of these isolation technologies, there still exist some problems, such as bulk chemical consumption, low isolation yield, low purity for final products, and harsh extraction conditions. Hence, seeking environmentally friendly, sustainable, and industrialized technologies to obtain the constituents in different biomass is still quite challenging to a large extent. In addition, due to the structural diversities of cellulose, hemicellulose, and lignin in different biomass, the modified constituents can be explored to prepare the diversified bio-materials and bio-chemicals for application in undiscovered bio-applications. Combining the new isolation, modification, and characterization technologies with novel applications will be the key steps to ultimately unlocking the integrated, economically viable, and multi-product biorefinery process.

The topic “*Isolation, modification, and characterization of the constituents (cellulose, hemicellulose, lignin, et al.) in Biomass and Their Bio-based Applications*” covers new pretreatment/fraction technologies for different biomass to achieve their valorization and bio-application of different substances in biomass. Here we sincerely thank all the contributors for their wonderful works on this topic. Following are the highlights drawn from their contributions to this special topic.

## New Methods to Prepare the Cellulose Derivatives From Different Biomass and Their Potential Applications

Liu et al., used carboxymethyl cellulose as the additive to improve the hydrophobicity and strength of carboxylated starch film, which is prepared from starch catalyzed by bio-α-amylase. They found that the addition of 15% carboxymethyl cellulose improved the mechanical properties of the prepared film with maximum tensile strength of 44.8 MPa. Carboxymethyl cellulose effectively improved the hydrophobicity of the starch film with the addition amount of 10%–30%, while hydrophobic property was stable at 66.8° when the addition amount was exceeded to 35%. Ou et al., isolated cellulose with high purity from waste rubber wood (RW) and further processed this into cellulose nanocrystals. They demonstrated that, compared with pure polylactic acid (PLA) film, the addition of acetylated RW cellulose reinforced the thermal properties of PLA composite films. The acetylated RW cellulose nanocrystals reinforced PLA composite films exhibited more enhanced performance in mechanical properties, in which highest tensile strength (55.0 MPa) and Young’s modulus (3.9 GPa) were achieved with 5% acetylated rubber wood cellulose nanocrystals. Yu et al., prepared a novel biosorbent from cellulose nanofibrils grafted with poly(m-aminobenzene sulfonate) (PABS) for effective detoxification and adsorption of Cr(VI) in an aqueous medium of 6,6-tetramethylpiperidine-1-oxyl (TEMPO)-oxidized cellulose nanofibrils (TOCNF). They demonstrated that the biosorbent from TOCNF-grafted PABS could detoxify and adsorb Cr(VI) synchronously.

## Environmentally Friendly, Sustainable, and Industrialized Technologies to Prepare Bio-Products From Different Biomass

Zhang et al., studied the inactivation of inulinase and marination of high-quality Jerusalem Artichoke (*Helianthus tuberosus* L.) pickles with screened dominant strains. They found that the enzymes in Jerusalem artichoke pickles were inactivated using a combination of NaCl and ultrasound exhibited better flavor than those exposed to NaHCO_3_. Further, this combination reduced the inulinase activity of the Jerusalem artichokes to 2.50 U/ml, which could maintain the inulin content at 61.22%. Yang et al., extracted and purified the natural rutin in *Sophora japonicato* to prepare fluorescent-responding sensor systems intended to recognize copper ions with both strong selectivity as well as appropriate sensitivity. They found that when rutin and (2-hydroxypropyl)-β-cyclodextrin were introduced within buffer solution, fluorescent emission intensity was significantly increased, in which the minimum detection limit can be 3.5 × 10^–8^ mol/L. Yang et al., prepared a powder consisting of the main components of bamboo shoots (cellulose, hemicellulose, and lignin) from fresh *Phyllostachys praecox* shoots as the supplement in the diet of mice, and aimed to evaluate the potential utility of these components as a dietary fiber supplement. They demonstrated that the major components of bamboo shoot powder (cellulose, hemicellulose, and lignin) could be used as beneficial natural additives in the food industry. Liu et al., used an enzyme at a 0.5% (w/w) loading dosage with the addition of ferulic acid esterase (1 U/g substrate) to obtain monosaccharides and ferulic acid from wheat bran autohydrolysis residues. The obtained hydrolysis yields were desirable, including 84.98% of glucose, 84.74% of xylose, 80.24% of arabinose, and 80.86% of ferulic acid. They demonstrated that a combination of autohydrolysis and enzymatic hydrolysis can achieve a satisfying yield of enzymatic saccharification and ferulic acid production from wheat bran. Cui et al., investigated the effects of microwave assisted liquid hot water (MA-LHW) pretreatment on the chemical composition of moso bamboo and the fiber structure of pretreated residues. They demonstrated that MA-LHW pretreatment increased the removal of hemicellulose, lignin, and other non-crystalline parts in bamboo materials, and more cellulose with crystalline structure was retained, which increased the CrI value of moso bamboo by 14.84%. Sun et al., developed a yeast strain of *Saccharomyces cerevisiae* A31Z for 2G bioethanol production from biomass. They found that this strain competed better than its ancestors in xylose utilization and subsequent ethanol production. In addition, this yeast strain manifested higher tolerance against common inhibitors from lignocellulosic hydrolysates, and it lowered the production of glycerol by-product. They provided a promising path for improving 2G bioethanol production in industries using *Saccharomyces cerevisiae*.

## Upgrading of Applications of Lignin

Yun et al., prepared the lignin fraction with a suitable structure to tailor excellent biological activities by sequentially organosolv fractionation with anhydrous acetone, 50% acetone, and 37.5% hexanes. They demonstrated that the lignin with high functional groups showed the ability to ameliorate *Escherichia coli*-induced diarrhea damage of mice to improve the formation of intestinal contents *in vivo*. Overall, this work demonstrated that a lignin fraction with a tailored structure can be used as a novel antimicrobial agent in the biomedical field. Fang et al., prepared magnetic lignin nanoparticles (MLN) from kraft lignin with Fe_3_O_4_ *via* Mannich reaction. They demonstrated that in terms of environmental protection and adsorption efficiency, MLN can act as an ideal adsorbent for Congo red dyes due to its simple preparation, superior performance, and convenient recovery. Yang et al., carried out different ball milling times on hardwood (poplar sawdust), softwood (larch sawdust), and gramineous material (bamboo residues) to understand the optimum condition to isolate the representative milled wood lignin (MWL) in these different biomass species. They demonstrated that milling time with 3 and 7 h were sufficient to isolate the representative lignin (with yield over 30%) in the cell wall of bamboo residues and poplar sawdust, respectively, while more than 7 h should be carried out to isolate the representative lignin in larch sawdust.

## Biological Technologies to Obtain the Constituents in Different Biomass

Li et al., focused on enzymatic hydrolysis of poplar sawdust xylan for production of XOS and xylose by a GH11 endo-1,4-β-xylanase MxynB-8 and a GH39 β-xylosidase Xln-DT. MxynB-8 showed the excellent ability to hydrolyze hemicellulose of broad leaf plants of poplar. Their results showed that the enzymatic hydrolysis yield of poplar sawdust xylan was improved by adding Xln-DT, and a xylose-rich hydrolysate could be obtained with the xylose yield of 89.9%. In addition, the enzymatic hydrolysis yield was higher (32.2%) by using MxynB-8 and Xln-DT together. Zhang et al., investigated the effects of short- and long-term cold stress (5°C) on the physiological characteristics, tissue-specific ginsenoside distributions, and ginsenoside synthesis gene expressions of 3-year-old *Panax ginseng* during the flowering period. They demonstrated that short-term cold stress can stimulate membrane lipid peroxidation, in turn stimulating the antioxidant enzyme system to alleviate oxidative damage and increase the expression of key enzyme genes involved in ginsenoside biosynthesis. Haq et al., reported dephenoliphication of wheat straw using various alkalis of Ca(OH)_2_ and NH_3_, acids of H_2_O_2_, H_2_SO_4_, and H_3_PO_4_, and combinations of NH_3_ + H_3_PO_4_ and H_3_PO_4_ + H_2_O_2_ at pilot scale to increase enzymatic saccharification yield. They found that upon subsequent saccharification of dephenoliphied substrate, the hydrolysis yield was recorded as 46.88%. Optimized conditions such as using 1% + 5% concentration of NH_3_ + H_3_PO_4_ for 30 min at 110°C temperature reduced total phenolic content (TPC) to 48 mg GAE/g DW. Zhao et al., proposed a recyclable and separable organic acid of furoic acid for hydrolyzing xylan to optimize XOS production by the response surface methodology. Their results indicated that the predicted maximum yield of XOS was 49.0% with 1.2% furoic acid at 167°C for 33 min, being close to the experimental value (49.2%). The results indicated that the fitted models were in good agreement with the experimental results to obtain the XOS. Liu et al., reported that *Saccharomyces cerevisiae* strain F106 could utilize xylose for ethanol production. Their results showed that the strains of F106-KR and diploid produced an ethanol yield of0.45 and 0.48 g/g total sugars, respectively, in simulated corn hydrolysates within 36 h. Using non-detoxicated corncob hydrolysate as the substrate, the ethanol yield with the triploid was approximately seven-fold more than that of the diploid at 40°C. Zhang et al., used dilute sulfuric acid pretreatment for tobacco stalk to enhance the performance of instant catapult steam explosion (ICSE). They found that the optimized 0.8% sulfuric acid (w/w) presoak–integrated ICSE pretreatment resulted in 85.54% nicotine removal from tobacco stalk. In addition, the total sugar concentration from enzymatic hydrolysis of pretreated tobacco stalk increased from 33.40 to 53.81 g/L; ethanol concentration increased 103.36% from 5.95 to 12.10 g/L in flask. They achieved the expected purpose of efficient utilization of discarded tobacco stalk. Boyi Ajeje et al., aimed to give an overview of the most recent thermostable cellulases and xylanases isolated from thermophilic and hyperthermophilic microbes. They emphasized on recent advancements in manufacturing these enzymes in other mesophilic hosts and the enhancement of catalytic activity as well as thermostability of thermophilic cellulases and xylanases, using genetic engineering as a promising and efficient technology for its economic production.

## Cellulose, Hemicellulose, and Lignin-Based Bio-Materials

Zhang et al., used the cellulose nanocrystal (CNC) suspensions with different concentrations (0.4%, 0.6%, and 0.8%) as the adjuvant to improve the dispersion ability of multilayer graphene (MLG) in aqueous suspension, which is easy to be aggregated by van der Waals force between layers. They found that CNC suspension with 0.8% concentration showed the highest ability to disperse 1.0 wt% MLG with the most stable performance in suspension. Sánchez et al., used the hemicellulose of O-acetyl galactoglucomannan (GGM) as the precursor material to prepare the hydrogels with ion exchange properties to remove Cu(II), Cr(VI), and As(V) ions. They found that the poly-GGM–glycidyl methacrylate-2-acrylamido-2-methyl-1-propanesulfonic acid hydrogel reached an absorption capacity of 90 mg/g for Cu(II). The poly-GGM–glycidyl methacrylate-(3-acrylamidopropyl)trimethylammonium chloride hydrogel reached values of 69 and 60 mg/g for Cr(VI) and As(V) oxyanions, respectively. Peng et al., used coaxial electrospinning technique to fabricate magnesium oxide (MgO) nanoparticles-incorporated PCL/gelatin core-shell nanocellulose periodontal membranes. They demonstrated that the incorporation of MgO nanoparticles barely affected the morphology and mechanical property of nanocellulose membranes and MgO nanoparticles-incorporated coaxial electrospinning PCL-derived nanocellulose periodontal membranes might have great prospects for periodontal tissue regeneration. Xu et al., used enzymatic hydrolysis residues (EHRs) of corncob residues to produce high lignin-containing cellulose nanofibrils (LCNFs) and lignin nanoparticles (LNPs) through a facile approach. They investigated morphology, thermal stability, chemical and crystalline structure, and dispersibility of the resultant LCNFs and LNPs. This work demonstrated that the lignocellulose-based nanomaterials with excellent properties could be achieved by coupling LCNFs and LNPs from EHRs. Huang et al., reviewed the recent developments and various applications of hemicellulose from wheat straw, including the hemicellulose-based derivatives and composites. In addition, they discussed the microstructure and molecule of hemicellulose extracted by different methods. Overall, this review will contribute to the development and high-value applications of hemicellulose from wheat straw.

